# Gut metabolomics profiling of non-small cell lung cancer (NSCLC) patients under immunotherapy treatment

**DOI:** 10.1186/s12967-020-02231-0

**Published:** 2020-02-03

**Authors:** Andrea Botticelli, Pamela Vernocchi, Federico Marini, Andrea Quagliariello, Bruna Cerbelli, Sofia Reddel, Federica Del Chierico, Francesca Di Pietro, Raffaele Giusti, Alberta Tomassini, Ottavia Giampaoli, Alfredo Miccheli, Ilaria Grazia Zizzari, Marianna Nuti, Lorenza Putignani, Paolo Marchetti

**Affiliations:** 1 Department of Clinical and Molecular Medicine, Sapienza University of Rome, Rome, Italy; 2 AOU Policlinico Umberto I, Rome, Italy; 3 Unit of Human Microbiome, Bambino Gesù Children’s Hospital, IRCCS, Rome, Italy; 4 Department of Chemistry, Sapienza University of Rome, Rome, Italy; 5 NMR-based Metabolomics Laboratory, Sapienza University of Rome, Rome, Italy; 6 Department of Medico-Surgical Sciences and Biotechnologies, Sapienza University of Rome, Rome, Italy; 7 AOU Sant’Andrea Hospital, Rome, Italy; 8 Department of Enviromental Biology, University of Rome, Rome, Italy; 9 Department of Experimental Medicine, University Sapienza, Rome, Italy; 10 Unit of Parasitology and Unit of Human Microbiome, Bambino Gesù Children’s Hospital, IRCCS, Rome, Italy

## Abstract

**Background:**

Despite the efficacy of immune checkpoint inhibitors (ICIs) only the 20–30% of treated patients present long term benefits. The metabolic changes occurring in the gut microbiota metabolome are herein proposed as a factor potentially influencing the response to immunotherapy.

**Methods:**

The metabolomic profiling of gut microbiota was characterized in 11 patients affected by non-small cell lung cancer (NSCLC) treated with nivolumab in second-line treatment with anti-PD-1 nivolumab. The metabolomics analyses were performed by GC–MS/SPME and ^1^H-NMR in order to detect volatile and non-volatile metabolites. Metabolomic data were processed by statistical profiling and chemometric analyses.

**Results:**

Four out of 11 patients (36%) presented early progression, while the remaining 7 out of 11 (64%) presented disease progression after 12 months. 2-Pentanone (ketone) and tridecane (alkane) were significantly associated with early progression, and on the contrary short chain fatty acids (SCFAs) (i.e., propionate, butyrate), lysine and nicotinic acid were significantly associated with long-term beneficial effects.

**Conclusions:**

Our preliminary data suggest a significant role of gut microbiota metabolic pathways in affecting response to immunotherapy. The metabolic approach could be a promising strategy to contribute to the personalized management of cancer patients by the identification of microbiota-linked “indicators” of early progressor and long responder patients.

## Background

Despite the efficacy of immune checkpoint inhibitors (ICIs) only 20–30% of treated patients present long term advantages [1].

Although immunohistochemistry detection of programmed death-ligand 1 (PD-L1) has been proposed as a predictive factor in both treatment of naïve and refractory non-small cell lung cancer (NSCLC) patients receiving pembrolizumab, atezolizumab or nivolumab, it presents several limitations such as expression dynamics, different antibody clones used and sampling choice [2].

Recently, tumor mutational burden (TMB) has emerged as an independent biomarker of ICIs outcomes across multiple cancer types, including NSCLC. Carbone et al. [1] recently showed that high TMB, calculated by whole exome sequencing (WES), is associated with improved overall response rate (ORR) (46.8% vs. 28.3%) and median progression-free survival (mPFS) [9.7 vs. 5.8 months; hazard ratio for disease progression or death (HR): 0.62; 95% confidence interval (CI), 0.38 to 1.00] in a group of untreated advanced NSCLC receiving nivolumab compared to platinum chemotherapy treated patients (Checkmate 026 Clinical Trial) [3], and it was proposed as biomarker in the Checkmate 227 study (ClinicalTrials.gov number, NCT02477826) [4]. However, TMB presented some limitation consisting for example in the cut-off score and in the technique adopted i.e., WES or Comprehensive Genomic Profiling (CGP). Furthermore, additional biomarkers are under investigations such as microsatellites [5], interferon signatures [6], T cell repertoire [7], major histocompatibility complex (MHC) status [8], and immune infiltrates [9].

In the context of multiple available biomarkers the changes occurring in the microbiota composition and metabolism have been proposed as a mechanism potentially affecting the response and the toxicity to immunotherapy [10].

Recently, several studies have confirmed the relevant role of the gut microbiota in the modulation of immune functions and its correlation with several diseases, including cancer [11]. In particular, gut–brain and gut–liver axes have already been investigated, while the relation between gut–lung axis has been newly suggested [12], in particular the hypothesis that changes in the gut microbiota could influence the lung microbiota, and vice versa (cross talk among microbial communities). However, the local and systemic influence of the gut microbiota, the influence on the lung microbiota and its products have not yet been fully assessed [13]. In fact, little is known about the hypothetical connection when looking at the world’s number one cause of death from cancer—lung cancer [14].

To date, the abundance of *Bifidobacterium* species (i.e., cocktail including *Bifidobacterium breve* and *Bifidobacterium longum*), has been observed to increase anti-tumor immunity and facilitate anti PD-L1 activity in germ free mice; moreover antibiotic treated mice presented impaired response to anti-Cytotoxic T-Lymphocyte Antigen 4 (CTLA-4) [15].

In melanoma patients treated with antiCTLA4 the presence of *Bacteroides* seems to have a protective role in terms of gastrointestinal toxicity [16].

Recently, it was also demonstrated that melanoma patients carrying gut microbiota enriched in *Faecalibacterium prausnitzii* presented longer progression free survival (lPFS) and overall survival (OS) [17], while in patients affected by NSCLC and renal cell carcinoma (RCC) patients treated with anti-PD-1 an higher distribution of *Akkermansia muciniphila* were assessed in responders compared with non-responders [18].

Despite promising results, the characterization of gut microbiota lacks full functional information about the relationship between the host–diet–microbiota axis, for this reason the study of metabolic profile of microbiota may actually provide new insights to overcome this gap [19].

Hence, as an effort to fill the knowledge gap, metabolomics enclose the comprehensive and concurrent systematic profiling of metabolic changes that occur in living systems in response to set of different factors as pathological, environmental or lifestyle conditions [20]. Metabolomics corroborate and enhances the information provided by genomics and proteomics [21] and have already shown promise in identifying metabolic phenotypes [22, 23].

In the present study, we aimed to characterize the metabolomic profile of NSCLC patients treated with nivolumab [2] and to investigate, for the first time, whether the gut microbiota metabolome may predict a baseline response to immunotherapy.

## Materials and methods

### Patient characteristics and sample collection

From April 2016 to March 2017 a cohort of 11 NSCLC patients aged 44 to 82 years (median age 68 years; 8 males and 3 females) were recruited at the Department of Clinical and Molecular Medicine, Sant’ Andrea Hospital, Sapienza University of Rome.

As inclusion criteria the following were considered: adult subjects, age > 18 years; NSCLC diagnosed by histology; Eastern Cooperative Oncology Group (ECOG) performance status ≤ 2; anti-PD-1 nivolumab employed as second-line treatment; acceptable pulmonary, cardiac, liver renal, and bone marrow functions.

As exclusion criteria the following were considered: autoimmune diseases; symptomatic interstitial lung disease and other noteworthy comorbidities; systemic immunosuppression; previous treatment with immune-stimulatory antitumor agents, including checkpoint-targeted molecules.

Nivolumab was proven at ordinary dosage of 3 mg/kg each 2 weeks till disease progression or undesirable toxicity. Radiological response was assessed with Response Evaluation Criteria in Solid Tumors (i-RECIST) Criteria and classified according to disease control (complete response, partial response and stable disease) and progressive disease. Toxicity was reported consistently with National Cancer Institute Common Terminology Criteria for Adverse Events (version 4.0) and toxicity valuation was performed at day 1 of every cycle until end of cure. The PFS was defined as the time from patient registration on clinical trial until the first documented tumor progression or death from any cause. The OS was defined as the time from patient registration to death from any cause. We defined as *early progressors* (EPs), patients experiencing disease progression within 3 months from the beginning of nivolumab, and *long responders* (LRs) patients presenting PFS longer than 12 months. The study was conducted according to good clinical practice guidelines and Helsinki declaration. The final version of the protocol was approved by the Institutional Ethics Committee (Ethical Committee n 4421, “Sapienza University”). Patients gave informed consent according to the guidance of the hospital ethics committee and with the approval of regulatory agencies. The fecal samples collected from NSCLC patients for gut microbiota metabolome profiling, were handled and processed for biobanking and integration processing at the OPBG Human Microbiome Unit and NMR-based Metabolomics Laboratory, Sapienza University of Rome.

### Targeted metagenomic on faecal microbiota

Genomic DNA from stool samples was manually extracted using QIAmp Fast DNA Stool mini kit (Qiagen, Germany), according to the manufacturer’s instructions.

Amplification of the variable region V3–V4 from the 16S rRNA gene (~ 460 bp) was carried out using the primer pairs described in the MiSeq rRNA Amplicon Sequencing protocol (Illumina, San Diego, CA).

The so obtained DNA amplicons were then cleaned-up by AMPure XP beads (Beckman Coulter Inc., Beverly, MA, USA). After second amplification step using a unique combination of bar-coded Illumina Nextera forward and reverse adaptor-primers, the final library was cleaned-up using 50 μL of AMPure XP beads and quantified using Quant-iT™ PicoGreen^®^ dsDNA Assay Kit (Thermo Fisher Scientific, Waltham, MA). Finally, the library was diluted in equimolar concentrations (4 nM), pooled together and sequenced on an Illumina MiSeqTM platform according to the manufacturer’s specifications.

Paired-ends reads were trimmed for their quality, read length and chimera presence using Qiime v1.8. pipeline [24]. Sequences were organized into Operational Taxonomic Units (OTUs) with a 97% of clustering threshold of pairwise identity and representative sequences were aligned using PyNAST v.0.1. software [25] against Greengenes 13_08 database [26] with a 97% threshold of similarity. Ecological and statistical analyses were performed using *phyloseq* and *DESeq2* packages from R software [27, 28].

### Gut microbiota metabolomics profiling

#### Generation of volatilome by gas chromatography solid-phase microextraction (GC–MS/SPME)

Fecal volatile organic compounds (VOCs) from 11 NSCLC patients were detected according to Vernocchi et al. [29] by using the carboxen–polydimethylsiloxane coated fiber (CAR-PDMS) (85 μm) and the manual SPME holder (Supelco Inc., Bellefonte, PA, USA) according to Vernocchi et al. [29]. The SPME fiber was exposed to each sample for 45 min. Both phases of equilibration and absorption were carried out under stirring condition. The fiber was then inserted into the GC injection port (10 min) for sample desorption and the GC–MS analyses carried out on an Agilent Technologies 7890B GC, coupled to a 5977A mass selective detector operating in electron impact mode (ionization voltage 70 eV), within a 1-mm quartz liner fitted system, equipped with a Supelcowax 10 capillary column (60 m length, 0.32 mm ID, Supelco, Bellefonte, PA, USA). The temperature program was the following: 50 °C for 1 min, 4.5 °C/min to 65 °C and 10 °C/min to 230 °C, which was held for 15 min. Injector, interface and ion source temperatures were 250, 250 and 260 °C. Total run time 35.83 min. The mass-scan range was 30–300 a.m.u. at 5.19 scans/s. Injections were carried out in splitless mode, under helium (1.5 mL/min) carrier. Molecule identification (ID) was carried out by using retention times (Rt) compared to pure compounds Rt (Sigma-Aldrich, Milan, Italy). The chromatograms were integrated and identified by comparing the fragment pattern with those in the mass spectral NIST library (version 2.2, NIST 14MS database; National Institute of Standards and Technology, Rockville, MD) and literature [30] followed by manual visual inspection. Quantitative compound data were expressed as ppm (mg/kg) obtained by interpolation of the relative areas vs. IS area.

### Determination of non-volatile metabolites by ^1^H-NMR spectroscopy

Fecal water was obtained as previously described [31]. After sample collection, 2 out of 11 NSCLC samples were excluded for insufficient sample collection. Briefly, ^1^H-NMR spectroscopy analysis was performed on 500 mg of feces suspended with 1 mL of D_2_O–PBS–NaN_3_ buffered solution. Each sample was vortexed for 2 min and then centrifuged for 25 min at 10,000 rpm and 4 °C to obtain fecal water. In total, 600 μL of supernatant was collected and analyzed according to Brasili et al. [31] NMR analysis was carried out at 298 K by using a Bruker Advance 400 spectroscope (Bruker BioSpin GmbH, Rheinstetten, Germany), equipped with a magnet operating at 9.4 Tesla (400.13 MHz for ^1^H frequency). 1D ^1^H NMR experiment was performed employing the standard presaturation *presat* pulse sequence. Spectral width was set to 6009 Hz (15 ppm) and 64 scans were collected for each spectrum with a presaturation pulse length of 2.00 s and a relaxation delay of 6.55 s. The spectra were collected with 64 K points for an acquisition time of 5.5 s [32]. The assignment of resonances was done by 2D homonuclear NMR Total Correlated Spectroscopy (TOCSY) and heteronuclear Single Quantum Coherence (HSQC) experiments. TOCSY experiments were recorded at 298 K with a spectral width of 15 ppm in both dimensions, using 8 K × 256 data points matrix, repetition time of 2 s and 80 scans with a mixing time of 110 ms. HSQC experiments were acquired with a spectral width of 12 ppm in proton dimension and 200 ppm in the carbon dimension, using 8 K × 256 data points matrix for the proton and the carbon dimensions, respectively, with a repetition delay of 2 s and 96 scans. The assignment was confirmed according to Human Metabolome Data Base [33] and own laboratory database. 1D ^1^H NMR spectra were processed and quantified by using the ACD Lab 1D-NMR Manager 12.0 software (Advanced Chemistry Development, Inc., Toronto, ON, Canada), whereas the MestReC software (Mestrelab Research SL, Santiago de Compostela, Spain) was used to assess 2D-NMR spectra. The quantification of metabolites was obtained by comparison of the integrals (normalized for number of protons) of specific signals with the IS trimethyl silyl propanoic acid (TSP) integral and then normalized for feces weight (expressed as µmol/g).

### Statistical analysis

To characterize the differences between NSCLC subjects from a single- and a multi-omic standpoint (i.e., ^1^H-NMR- and GC–MS-based metabolomics), a data analytical strategy based on the use of multivariate chemometric methods were carried out on the integrated dataset of 9 patients considering both GC–MS and ^1^H-NMR data. However, in order to have reliable results, prior to data processing, the raw data matrix of GC–MS/SPME metabolites was cleaned by retaining only those molecules which were detected in at least 80% of the investigated subjects. Such a screening was not needed in the case of ^1^H-NMR data. Since the two blocks of data came from different experimental techniques and had different variances and because of high inherent variability, data were block scaled after individual autoscaling. Metabolomics multivariate data analysis was carried out using *in house* written functions running under MATLAB (R2015b; The Mathworks, Natick, MA) environment. Principal components analysis (PCA) was used to analyze inherent clustering, to identify outliers and significant metabolites. Mann–Whitney U test was then applied to assign significant differences at the level of single metabolite in the PCA model in particular, a *p*-value of 0.05 was considered as threshold for statistical significance.

## Results

### Clinical characteristics

Eleven patients with stage IV NSCLC treated with second-line nivolumab were enrolled in this study. Baseline clinical characteristics of patients are summarized in Table 1. Among them, 10 patients had squamous-cell carcinoma and one adenocarcinoma. Median PFS and OS were 7.5 and 7.7 months, respectively. Four patients were EPs, while 7 LRs (Table 1).

**Table 1:** Clinical features of NSCLC patients: phenomic metadata

Patient characteristics at baseline	N° (%)
Age	
≤ 65	5 (45)
> 65	6 (55)
Sex	
Male	8 (73)
Female	3 (27)
Histology	
Adenocarcinoma	1 (10)
Squamous cell carcinoma	10 (90)
ECOG performance status	
0–1	10 (90)
> 1	1 (10)
N° sites of metastasis	
1	2 (18)
> 1	9 (82)
Brain metastases	
Present	1 (10)
Absent	10 (90)
Treatment line	
2	11 (100)
> 2	0 (0)
Previous platinum based chemotherapy	
Yes	9 (82)
No	2 (18)
Response to nivolumab	
Early progressors (EPs)	4 (36)
Long responder (LRs)	7 (64)

### Gut microbiota ecological analysis

The metagenomics data have been analyzed in a preliminary way and no cluster formation has been highlighted between EPs and LRs in the structure of microbiota (Additional file 1: Figure S1).

In particular, the analysis performed by Bray–Curtis dissimilarity (Additional file 1: Figure S1, panel A) does not highlight the existence of a specific cluster related to the clinical condition of EP or LR. Furthermore, this is confirmed in the distribution of the main bacterial genera for each sample (Additional file 1: Figure S1, panel B).

### Gut microbiota metabolomics

Each fecal sample was analysed to determine both VOCs and non-volatile metabolites, in an untargeted fashion, thus capturing large numbers of known and uncharacterized metabolites, including those of potential microbial origin.

### VOCs profiling

Two-hundred and twenty-three VOCs for all 11 NSCLC patients were identified and quantified by GC–MS/SPME, and grouped into the following 18 chemical classes: alcohols (n 44); esters (n 31); aldehydes (n 21); ketones (n 35); alkenes (n 12); alkanes (n 17); acids (n 8); phenols (n 4); terpenes (n 18); sulfur compounds (n 2); hydrazine (n 1); azetidine (n 1); indoles (n 7); pyridine (n 1); amines (n 13); furans (n 2); pyrazine (n 2) and aromatic hydrocarbons (n 4) (data not shown). After data reduction, a matrix with 24 metabolites detected in the 80% of samples was considered for computation (Additional file 2: Table S1).

### Non-volatile metabolite profiling

Fourty-nine non volatile metabolites were detected (i.e., acids, amino acids, amines, and sugars) and quantified (µmol/g) by ^1^H-NMR for 9 NSCLC patients (Additional file 3: Table S2).

### Integrated metabolomic model

**Fig. 1:**
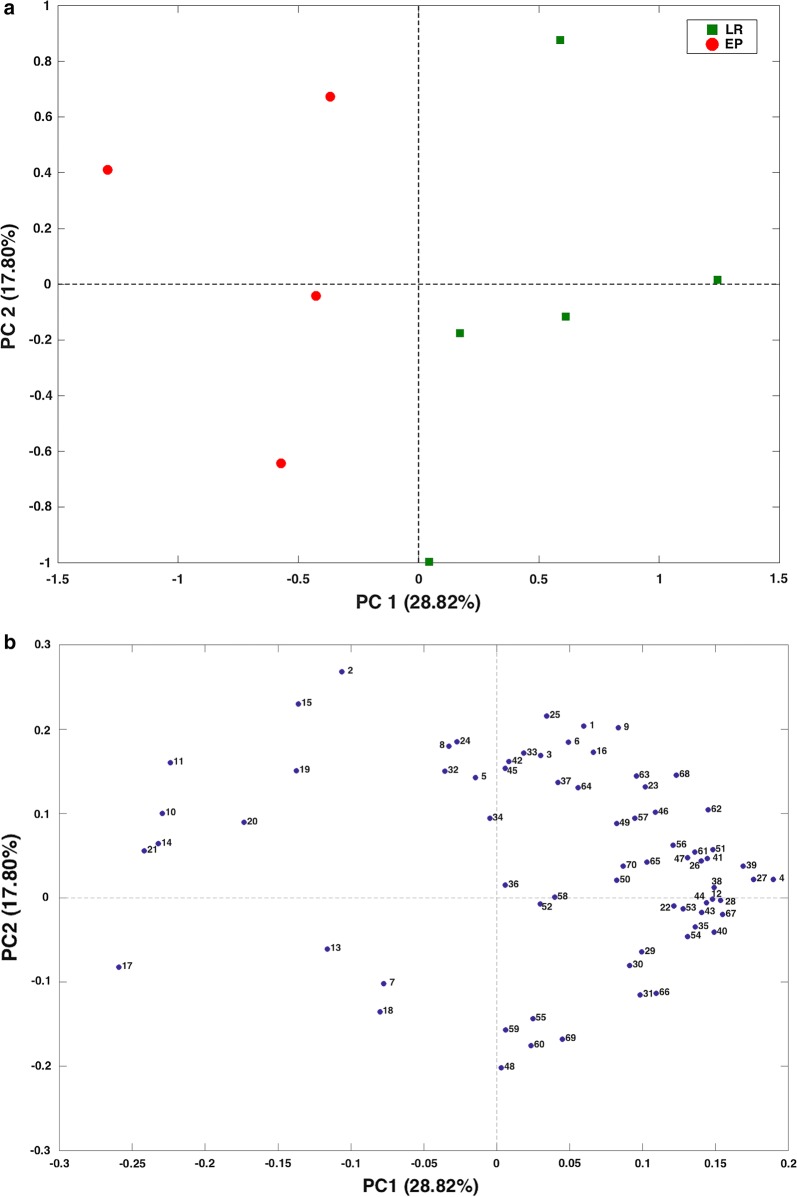
Principal component analysis (PCA) of integrated datasets of ^1^H-NMR and GC–MS/SPME data at T_0_. **a** PC score plot. **b** Loading plot. The first two components explained 47% of the total variance. In green and red circles are represented EP (early progressors, not responders) and LR (long responders) patients, respectively. **a** Red, early progressors (EPs); green, long responders (LRs). **b** 1: 1-butanol; 2: 1-hexanol; 3: 1-pentanol; 4: 2,6-dimethyl 4 heptanone; 5: 2-butanone; 6: 2-heptanone; 7: 2-hexanol; 8: 2-nonanone; 9: 2-octanol; 10: 2-octanone; 11: 2-pentanone; 12: 6-methyl-5-hepten-2-one; 13: benzaldehyde; 14: benzeneacetaldehyde; 15: *cis*-2,6-dimethyl-2,6-octadiene; 16: dimethyl disulfide; 17: dodecane; 18: indole; 19: methyl isobutyl ketone; 20: *p*-cresol; 21: tridecane; 22: Bile salt 1; 23: Bile salt 2; 24: U1; 25: 2-hydroxy-3-methylbutyric acid 26: U2; 27: valeric acid; 28: isovaleric acid; 29: leucine; 30: valine; 31: isoleucine; 32: U3; 33: 2-oxoisovaleric acid; 34: ethanol; 35: lactic acid; 36: acetoin; 37: 2-aminoisobutyrate; 38: alanine; 39: butyric acid; 40: lysine 41: acetic acid; 42: *N*-acetyl-moieties; 43: propionic acid; 44: glutamic acid; 45: succinic acid; 46: U4; 47: methionine; 48: aspartic acid; 49: trimethylamine (TMA); 50: 2-oxoglutarate; 51: malonic acid; 52: U5; 53: choline; 54: taurine; 55: methanol; 56: glycine; 57: b-arabinose; 58: b-galactose; 59: b-xylose; 60: b-glucose; 61: U6; 62: uracil; 63: orotic acid; 64: U7; 65: fumaric acid; 66: tyrosine; 67: phenylalanine; 68: U8; 69: formic acid; 70: nicotinic acid

In order to have a multi-omics overview, the integration of GC–MS/SPME and ^1^H-NMR data was carried out through a low-level data fusion approach. Therefore, PCA was applied to the matrix obtained by concatenation of GC–MS/SPME and ^1^H-NMR data after block-scaling. The PCA scores plot (PC1 vs. PC2; PC1: 28.82%; PC2: 17.80%), displayed in Fig. 1, panel a, showed a clear separation between EP and LR patients (Fig. 1, panel a). In particular, the separation between the two groups occurred mainly along the axis of PC1; therefore, in order to assess which metabolites mainly contributes to the observed separation, only the variables having the highest absolute value of the loadings on such component were considered (Fig. 1, panel b).

Accordingly, inspection of the loadings plot allowed observing how the gut microbiota metabolome of LRs patients was mostly characterized by SCFAs (i.e., butyric, valeric, acetic and propionic), AAs (i.e., lysine) and nicotinic acid. On the contrary, EPs were mainly represented by alcanes (i.e., tridecane, dodecane) ketones (i.e., 2-pentanone, 2-octanone), aldehydes (i.e., benzeneacetaldehyde), and *p*-cresol. For glutamic acid, isoleucine, 2-octanone, valeric acid, acetic acid, butyric acid and dodecane the level differences between EPs and LRs subjects were not statistically significant (data not shown).

**Fig. 2:**
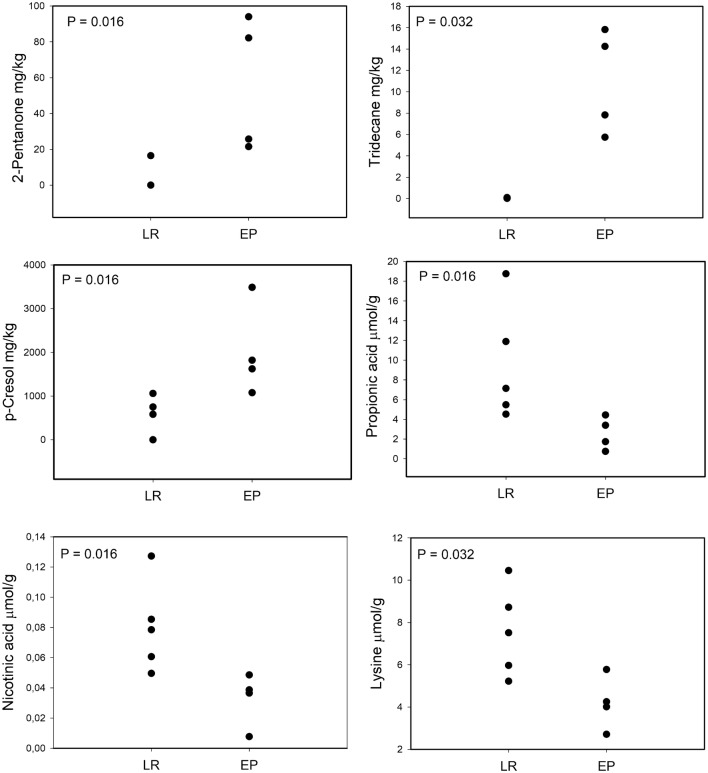
Concentration (µmol/g) of proprionic acid (median LR = 7.14 EP = 2.56), nicotinic acid (median LR = 0.08 EP = 0.04), lysine (median LR = 7.51 EP = 4.13), 2-pentanone (median LR = 0; EP = 53.9) tridecane (median LR = 0; EP = 11.03) and *p*-cresol (median LR = 582.91; EP = 1721.48)

Interestingly, the levels of tridecane could be measured only in fecal metabolome of EPs patients, being absent in that of LR ones. Particularly, the differences between levels of propionic acid (p value = 0.016), lysine (p value = 0.032), nicotinic acid (p value = 0.016), tridecane (p value = 0.032) 2-pentanone (p value = 0.016) and *p*-cresol (p value = 0.016) were statistically significant, as inferred by U-Mann–Whitney test. PCA model took into account the covariance of propionic acid with the other SCFAs (Fig. 2).

## Discussion

Some evidences suggest that gut microbiota-induced immune effects dependent on the specific therapy for different type of cancer [15, 34, 35].

Chemotherapy could impact on both immune system and gut microbiota, influencing the relationship between both of them [14].

By metagenomics approach no statistical significant association were found between EPs and LRs; moreover it was not possible to hypothesize a potential relationship with the clinical outcomes.

Untargeted metabolomics may contribute to highlight gut microbiota metabolites allowing to infer host-microbiome co-metabolism [36]. The application of multivariate chemometric model based on the GC–MS and ^1^H-NMR data may concur to clarify the responsiveness to immune therapy of NSCLC patients under specific disease conditions.

The gut microbiota metabolome in cancer has been particularly investigated to give information on the relationship between colorectal cancer and gut microbiome. However, the role of specific bacteria metabolism in carcinogenesis and progression remains an active area of inquiry [37].

Metabolites and VOCs have been investigated in lung cancer on exhaled breath [38–40] and urine [41, 42]. Therefore, low weight molecules have been proposed as biomarkers of lung cancer. Up to date the interindividual variations of gut microbiota composition have been studied to find a possible relationship between enterotype and immune-checkpoint therapy response in NSCLC patients [14, 18]. In the present study, by using a metabolomics approach, was detected for the first time a gut microbiota metabolome potentially associated to NSCLC patients responsiveness to immune checkpoint inhibitors (ICIs). Fecal samples of EPs NSCLC patients were characterized by a metabolic profile constituted by high levels of alkanes (tridecane and dodecane), methylketones (2-pentanone and 2-octanone), *p*-cresol, and low levels of SCFAs (propionic, butyric, acetic, valeric acids), aminoacids (lysine, isoleucine and glutamic) and nicotinic acid suggesting an imbalanced microbiota metabolism in agreement with a dysbiotic intestinal microbiota as reported in previous studies [18]. Patients affected by NSCLC with longer progressive free-survival have an higher abundance of *Akkermansia muciniphila* and of other commensales such as *Ruminococcus* spp., *Alistipes* spp., and *Eubacterium* spp., with a relative under representation of *Bifidobacterium adolescentis*, *B. longum*, and *Parabacteroides distasonis* [18]. *Akkermansia muciniphila* is a mucin degrader producing propionate through propanediol pathway, as well as some of *Ruminococcus* spp. fermenting fucose, released from mucin degradation, for the propionate production [43]. Generally, SCFAs reach very high concentrations in the colon [44], reducing pH, satisfying nutritional requirements, microbial function regulation and composition, and also affecting the immune system [45]. SCFAs regulate innate immune cells such as neutrophils acting as neutrophil chemotaxin, macrophages and dendritic cells (DCs) by G-protein-joined receptors (GPCRs) and HDACs and also modulate bidirectionally antigen-specific adaptive immunity mediated by T-cells and B-cells.

In fact, SCFAs regulate the immune system by histone deacetylases (HDACs), receptors and/or metabolic integration.

Heerdt et al. [46] highlighted that SCFAs are generally perceived as tumor suppressors as they induce cancer cell differentiation and apoptosis. Acetic and butyric acid levels were reduced in patients with colitis, colon cancer, or other intestinal disorders as inflammatory bowel disorder [47, 48].

The gut–lung axis theory [49] suggests that microbes or their metabolic products might have systemic effects, and hence, could give an effect on the lung bacterial composition and immune response. For this reason, fecal SCFAs, which are considered immune modulators/protectors of the intestinal barrier [50], might be possible mediators of the gut “long-distance” affecting directly or indirectly the target site by the stimulation of gut/circulating immune system [14]. Therefore, the gut microbiota of EPs patients appears characterized by a low metabolic activity producing SCFAs associated to an high production of *p*-cresol as compared to LRs. *P*-cresol is a microbial metabolite produced from tyrosine through tyrosine lyase or tyrosine aminotransferase β activity; *p*-cresol producers belonged to the *Coriobacteriaceae* and *Clostridium* clusters XI and XIVa [51]. The *p*-cresol exhibits cytotoxicity and genotoxicity and reduces endothelial barrier function in vitro [52, 53]. *P*-cresyl sulfate, a sulfate-conjugate of *p*-cresol, suppresses Th1-type cellular immune responses in mice [39, 42]. High levels of this metabolite in urine have been found in patients with cardiovascular and renal diseases [51]. Finally, low levels of nicotinic acid (niacin) complete the metabolic profile of EPs patients. In general, the niacin levels in the feces depend on the food intake, however gut bacteria metabolism could also contribute to the fecal levels of this metabolite [54]. Niacin is a important precursor for the synthesis of nicotinamide adenine dinucleotide (NAD+) that is the central cofactor of metabolism, mediating ATP generation, energy substrate oxidation, reactive oxygen species (ROS) detoxification, DNA repair, and nutritionally sensitive gene regulation [55].

Indeed, in general, metabolome encloses the comprehensive systematic profiling of metabolic changes that occur in living systems in response to sets of different factors such as pathological, environmental or lifestyle conditions [56]. Gut microbiota metabolomic profile corroborates and enhances the information provided by genomics and proteomics [57] and has already shown promises in identifying metabolic phenotypes associated to microbiota patterns [22].

## Conclusion

In our study SCFAs mainly characterize the gut microbiota metabolome of LRs subjects to immune therapy and therefore it can be hypothesized that these molecules might be considered as biomarker of responsiveness. On the contrary, the fecal levels of tridecane might be considered as biomarker of non-responsiveness.

These results should be of support to better understand the close interaction between the gut microbiota of different “communicating” body districts and their interaction with the immune system in NSCLC patients. The power of this study approach is that data collection through non-invasive techniques can be incorporated into standard laboratory exams, hence modulating treatment of the patients.

However, it is necessary to pursue with larger follow-up clinical studies, in order to provide more representative datasets. The mechanism how these microbial metabolites alone or in combination modulate the host immune system remains to be highlighted. Hence, the identification of biomarkers might help in designing new personalized or “alternative” therapies in lung cancer treatment, with also a better characterization of patient’s status and diagnosis, as well as in searching new ways to improve immunotherapy tolerance and immune therapies responses, also implemented with nutritional support as pre, pro, postbiotics and symbiotics.

## Supplementary information

**Additional file 1: Figure S1.** Panel A. Analysis of Bray-curtis of the analyzed samples divided by EP (blue circle) and LR (yellow triangle). Panel B. Distribution of the relative abundances of bacterial genera present in addition to 0.01% for each sample in the two groups considered EP and LR.

**Additional file 2: Table S1.** GC-MS/SPME metabolites raw data.

**Additional file 3: Table S2.**^1^H-NMR metabolites raw data.

## Data Availability

All data generated and analyzed during the current study are included in this published article.
