# Supplementary figures and images for: Gut metabolomics profiling of non-small cell lung cancer (NSCLC) patients under immunotherapy treatment
Source: J Transl Med. 2020 Feb 3;18:49. doi: 10.1186/s12967-020-02231-0 (PMC6998840; doi:10.1186/s12967-020-02231-0)

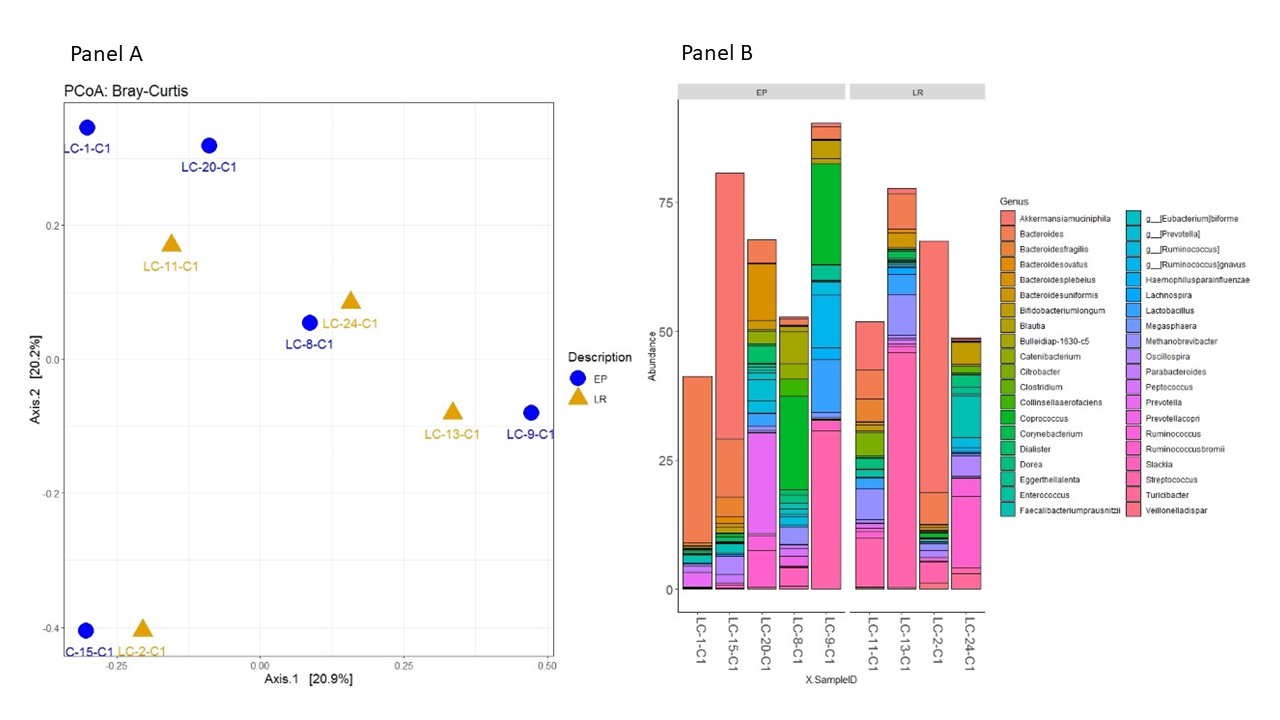

Supplement: Supplementary file 1 — Additional file 1: Figure S1. Panel A. Analysis of Bray-curtis of the analyzed samples divided by EP (blue circle) and LR (yellow triangle). Panel B. Distribution of the relative abundances of bacterial genera present in addition to 0.01% for each sample in the two groups considered EP and LR. [file 12967_2020_2231_MOESM1_ESM.tif]
